# Persistent Pediatric Cervical Lymphadenopathy: A Case Report of Kikuchi–Fujimoto Disease Mimicking Infection and Malignancy

**DOI:** 10.1155/crpe/4898588

**Published:** 2026-10-27

**Authors:** Maegan Thomas, Dillon Stevens, Chloe Davis, Nathan M. Money

**Affiliations:** ^1^ Spencer Fox Eccles School of Medicine University of Utah Health Salt Lake City Utah USA; ^2^ Department of Pediatrics University of Utah Health Salt Lake City Utah USA

**Keywords:** case report, cervical lymphadenopathy, histiocytic necrotizing lymphadenitis, Kikuchi–Fujimoto disease, pediatric

## Abstract

**Case Presentation:**

A 10‐year‐old female presented with 2 months of unilateral cervical lymphadenopathy despite several courses of oral antibiotics. She presented to the emergency department due to persistent pain and swelling along with new fever. On presentation, she was febrile to 39.0°C. Physical examination revealed significant left‐sided cervical and submandibular lymphadenopathy with tenderness, warmth, and overlying erythema. Initial labs were notable for leukopenia (2.6 × 10^3^/μL), thrombocytopenia (135,000/μL), and elevated liver enzymes. Computed tomography (CT) of the neck demonstrated necrotic left cervical lymphadenopathy with prominent right cervical lymph nodes. An infectious workup, including testing for Epstein–Barr virus, *Cytomegalovirus*, human immunodeficiency virus, coccidioidomycosis, tuberculosis, and microbial cell‐free DNA sequencing, was negative. Initial fine‐needle aspiration (FNA) and core needle biopsy showed predominantly necrotic tissue. Cultures obtained during the biopsy showed no growth, and flow cytometry was negative for leukemia/lymphoma markers. Intravenous (IV) ceftriaxone administration resulted in improvement in neck swelling and fever resolution. Histopathologic examination from excisional biopsy revealed histiocytic necrotizing lymphadenitis consistent with Kikuchi–Fujimoto (KFD) lymphadenitis. Comprehensive autoimmune serologies were negative. Naproxen was prescribed to help with residual inflammation.

**Discussion:**

KFD should be considered in children presenting with prolonged cervical lymphadenopathy refractory to antibiotics. This case illustrates several diagnostic challenges inherent in making this diagnosis. The patient’s prolonged unilateral cervical lymphadenopathy, necrotic imaging findings, and transient improvement with antibiotics strongly suggested bacterial lymphadenitis or malignancy. However, the persistence of symptoms despite multiple appropriate antibiotic courses, the development of cytopenias and elevated liver enzymes, and ultimately nondiagnostic cultures represented key findings prompting reconsideration of the initial differential diagnoses. This case also highlights the importance of considering excisional biopsy when core biopsy is nondiagnostic.

## 1. Introduction

Kikuchi–Fujimoto disease (KFD) is a rare, self‐limited inflammatory disorder characterized by histiocytic necrotizing lymphadenitis [1] and most commonly presents in adolescents and young adults with fever and subacute, tender cervical lymphadenopathy [2]. KFD carries substantial diagnostic overlap with infectious, malignant, and autoimmune etiologies, often resulting in delayed diagnosis and extensive diagnostic evaluation [3, 4]. Diagnosis of KFD is challenging because cervical lymph node fine‐needle aspiration (FNA) and core needle biopsy are often nondiagnostic due to extensive necrosis and sampling limitations [4, 5]. Excisional lymph node biopsy remains the gold standard for diagnosis, with characteristic histopathologic findings including necrosis with karyorrhectic debris, histiocytic infiltrates, and absence of neutrophils [1, 3–6].

We report a case of a 10‐year‐old female with persistent cervical lymphadenopathy despite five courses of antibiotics, whose clinical and imaging findings initially raised concern for persistent infection or malignancy. Extensive infectious and oncologic evaluation and initial needle biopsy failed to establish a diagnosis, which was ultimately made by excisional lymph node biopsy. This case illustrates how pediatric KFD can closely mimic more common causes of cervical lymphadenopathy and highlights the importance of reconsidering KFD when lymphadenopathy persists despite antimicrobial therapy and minimally invasive biopsy is nondiagnostic.

Written informed consent for publication of this case report and accompanying images was obtained from the patient’s parent/legal guardian. This submission was discussed with the University of Utah Institutional Review Board, which felt it did not constitute human subjects research and therefore was not subject to the review process.

## 2. Case Presentation

A previously healthy, 10‐year‐old White female with Asian ancestry through her maternal grandmother presented with a 2‐month history of progressive unilateral cervical lymphadenopathy. Her symptoms began following travel to Oregon and a viral illness within the household. Over time, she developed persistent tenderness and swelling of the left neck with waxing and waning dull and stabbing pain. She was treated empirically for presumed bacterial cervical lymphadenitis with five separate courses of oral antibiotics over months. Although there was no sustained improvement, intermittent partial improvement in lymphadenopathy during antibiotic therapy raised concern for an incompletely treated bacterial infection and contributed to continued antimicrobial treatment. She subsequently developed high‐grade fevers, headache, nausea, vomiting, and decreased oral intake, prompting evaluation in the emergency department.

On presentation to the emergency department, she was febrile to 39.0 C (102.2°F) with otherwise stable vital signs. Physical examination demonstrated significant left‐sided cervical and submandibular lymphadenopathy (Figure 1) with tenderness to palpation and restricted neck movement. Laboratory evaluation revealed leukopenia (white blood cell count 2600 per μL), thrombocytopenia (135,000 per μL), and elevated transaminases (AST 103 U/L and ALT 101 U/L). Computed tomography (CT) of the neck demonstrated necrotic left cervical lymphadenopathy with prominent right cervical lymph nodes.

**FIGURE 1:**
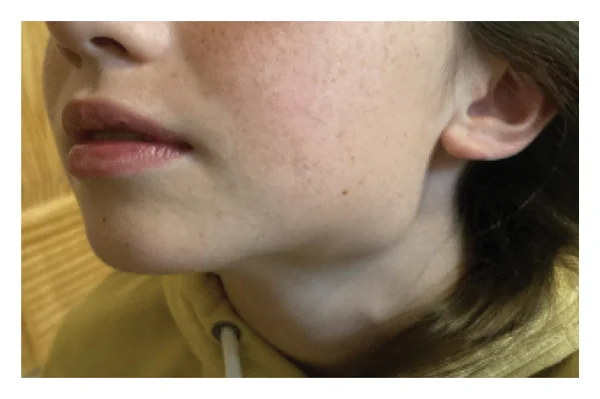
Initial patient presentation to the ED. The image shows left cervical lymphadenopathy.

Further history revealed extensive animal exposures, including cows, cats, dogs, horses, geese, and ducks on the family farm, as well as recent consumption of raw milk. Given her prolonged clinical course and exposure history, pediatric infectious disease was consulted. An extensive infectious evaluation, including testing for Epstein–Barr virus, *Cytomegalovirus*, human immunodeficiency virus, coccidioidomycosis, tuberculosis, and microbial cell‐free DNA sequencing testing (Karius Spectrum), was unrevealing. FNA and core needle biopsy demonstrated predominantly necrotic tissue without organism growth on culture, while flow cytometry showed no abnormal myeloid, B‐cell, T‐cell, natural killer cell, or plasma cell populations. During hospitalization, she received two doses of intravenous (IV) ceftriaxone, after which her fever resolved and cervical swelling improved. Antibiotics were subsequently discontinued, and she was discharged with close outpatient follow‐up.

Because the initial biopsy was nondiagnostic, an excisional lymph node biopsy was performed 2 days later. Excisional lymph node biopsy demonstrated preserved nodal architecture with focal necrosis (Figure 2a), expansion of the interfollicular regions by histiocytes and immunoblasts (Figure 2b), and areas of necrosis without neutrophilic infiltration surrounded by crescentic histiocytes (Figure 2c). Immunohistochemical staining demonstrated a T‐cell–predominant lymphocyte population (CD3) (Figure 3b), with CD123 highlighting occasional plasmacytoid dendritic cells (Figure 3a). These findings, in conjunction with the characteristic histopathologic features, supported the diagnosis of KFD. Epstein–Barr virus in situ hybridization, acid‐fast bacilli, and Grocott–Gomori methenamine silver stains were negative. Histiocytic necrotizing lymphadenitis on histology raised concern for either lupus lymphadenitis or KFD. Pediatric rheumatology was consulted, and comprehensive autoimmune serologic testing, including antinuclear antibody, antidouble‐stranded DNA antibody, complement levels, and other lupus‐specific antibodies, was negative. These findings excluded systemic lupus erythematosus (SLE) at the time of diagnosis and supported a diagnosis of KFD. The patient was treated with naproxen for symptomatic relief of residual inflammation. A complete detailing of the patient’s clinical course can be found in Table 1.

**FIGURE 2:**
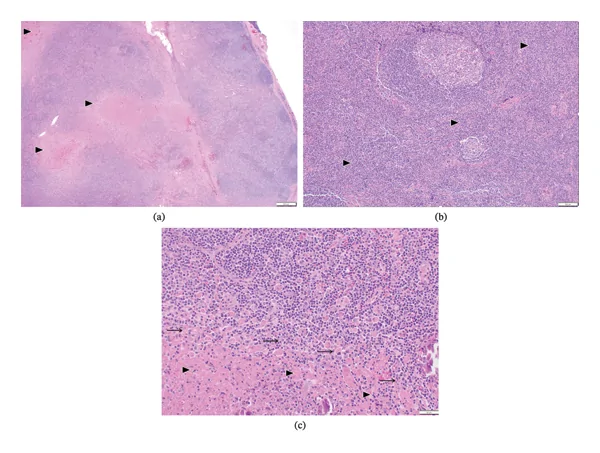
(a) Hematoxylin and eosin (H&E) stain demonstrating a low‐power view of the left neck mass. The mass shows a lymph node displaying retained lymph node architecture with retained germinal centers, patent sinuses, and intact capsule. There are foci of architecture disrupted by necrosis (arrowhead). The image was captured with a 2x objective. (b) Hematoxylin and eosin (H&E) stain demonstrating an intermediate‐power view of the left neck mass. The mass shows a lymph node displaying retained lymph node architecture with retained germinal centers, patent sinuses, and intact capsule. Interfollicular areas are expanded by a polymorphous population (arrowhead) composed of increased immunoblasts, histiocytes and small lymphocytes. The image was captured with a 10x objective. (c) Hematoxylin and eosin (H&E) stain demonstrating an intermediate‐power view of the left neck mass. The mass shows a lymph node displaying retained lymph node architecture with retained germinal centers, patent sinuses, and intact capsule. There are foci of architecture disrupted by necrosis without neutrophils (arrowhead), surrounded by an abundant histiocytic infiltrate including occasional crescentic (or twisted) histiocytes (arrow). The image was captured with a 20x objective.

**FIGURE 3:**
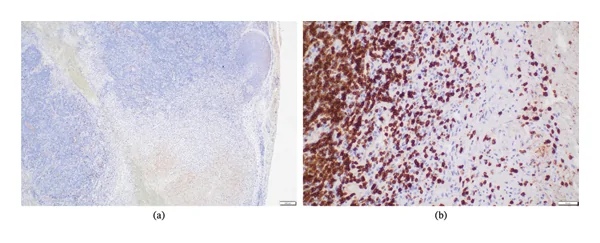
(a) Cluster of differentiation 123 (CD123) stain highlighting occasional plasmacytoid dendritic cells. Image was captured with a 4x objective. (b) Cluster of differentiation 3 (CD3) stain highlighting T‐cell–predominant lymphocyte population. The image was captured with a 20x objective.

**TABLE 1:** Timeline of the patient’s clinical course, diagnostic evaluation, and management.

Timeline	Clinical events, investigations, treatments
∼2 months before admission	Developed progressive, tender left cervical lymphadenopathy following travel to Oregon and a viral illness within the household
Following 2 months	Persistent lymphadenopathy despite five courses of oral antibiotics
3 days before admission	Developed worsening cervical pain, headache, malaise, and systemic symptoms
Day of admission	Developed high‐grade fever, nausea, vomiting, and decreased oral intake. Emergency department evaluation showed WBC 2600/μL, platelets 135,000/μL, aspartate aminotransferase (AST) 103 U/L, and alanine aminotransferase (ALT) 101 U/L. CT neck demonstrated necrotic left cervical lymphadenopathy with prominent right cervical nodes
Hospitalization	Extensive infectious evaluation was unrevealing. FNA and core needle biopsy yielded predominantly necrotic tissue; cultures showed no growth and flow cytometry showed no abnormal cell populations. Received two doses of IV ceftriaxone with resolution of fever and improvement in cervical swelling. Antibiotics subsequently discontinued
2 days after discharge	Excisional lymph node biopsy was performed because the initial biopsy was nondiagnostic. Histopathology demonstrated histiocytic necrotizing lymphadenitis concerning for KFD versus lupus lymphadenitis
Following biopsy	Rheumatologic evaluation including antinuclear antibody (ANA), anti‐dsDNA antibodies, complement levels, and other lupus‐specific antibodies was negative, supporting Kikuchi–Fujimoto disease. Naproxen was prescribed for residual inflammation

## 3. Discussion

KFD is an uncommon but important cause of prolonged cervical lymphadenopathy in pediatric patients. Although its etiology is not fully understood, current evidence suggests it may result from an exaggerated T‐cell–mediated immune response to infectious agents in genetically susceptible individuals [1, 4, 5]. Viral pathogens including Epstein–Barr virus, *Cytomegalovirus*, *Parvovirus* B19, and human herpesvirus 6 have been proposed as potential triggers, although no single causative organism has been established [2, 3, 7]. KFD occurs worldwide but most commonly affects adolescents and young adults, with pediatric cases increasingly being recognized [1, 2]. Subacute cervical lymphadenopathy and fever remain the predominant presenting manifestations, while leukopenia is among the most frequently reported laboratory abnormalities [1–3, 7].

Our patient’s presentation shared several features previously described in pediatric KFD, including fever, unilateral cervical lymphadenopathy, leukopenia, thrombocytopenia, and transaminitis. In a pediatric series of 16 patients, Park et al. reported leukopenia in 87% and mild transaminase elevation in 43.8% of patients; notably, half were diagnosed with an infectious illness and treated with antibiotics [8]. Larger pediatric cohorts have similarly identified cervical lymphadenopathy and fever as the predominant presenting features, with leukopenia occurring frequently and thrombocytopenia less commonly [9]. Thus, although the individual clinical and laboratory abnormalities in our patient were consistent with previously reported pediatric KFD, the prolonged clinical course and persistence of lymphadenopathy despite five antibiotic courses contributed to continued concern for infection or malignancy and delayed recognition of KFD.

The differential diagnosis of persistent cervical lymphadenopathy in children is broad and includes bacterial lymphadenitis, atypical mycobacterial infection, tuberculosis, cat scratch disease, viral infections, lymphoma, and autoimmune diseases such as SLE [1, 6]. In our patient, necrotic cervical lymphadenopathy on CT, high‐grade fever, and cervical tenderness initially supported concern for an infectious process, while the prolonged course and associated cytopenias also warranted evaluation for malignancy. Importantly, nodal necrosis is not specific to infection or malignancy and has also been described in pediatric KFD [10]. In one pediatric imaging series, nonenhancing necrosis was identified in 63% of affected cervical lymph nodes, illustrating the substantial radiographic overlap between KFD and other causes of necrotic cervical lymphadenopathy [10].

Tissue diagnosis of KFD can be difficult because FNA and core needle biopsy frequently yield nondiagnostic results when extensive necrosis limits evaluation of lymph node architecture [1, 5]. In our patient, FNA and core needle biopsy yielded predominantly necrotic tissue without definitive diagnostic features, while flow cytometry showed no abnormal lymphoid or myeloid populations. Because minimally invasive sampling was nondiagnostic, excisional lymph node biopsy was pursued and demonstrated histiocytic necrotizing lymphadenitis characterized by necrosis without neutrophilic infiltration and surrounding histiocytes, including crescentic histiocytes. Immunohistochemical findings demonstrating a T‐cell–predominant lymphoid population with B cells retained in a typical follicular distribution provided additional evidence against lymphoma. Excisional biopsy remains the diagnostic gold standard when KFD is suspected despite inconclusive minimally invasive sampling [1, 5].

Distinguishing KFD from SLE lymphadenitis is particularly important because the two conditions share overlapping clinical and histopathologic features including fever, cervical lymphadenopathy, cytopenias, and necrotizing lymphadenitis [11]. Lupus lymphadenitis is more commonly associated with positive autoimmune serologies, hypocomplementemia, and hematoxylin bodies on histopathology [11, 12]. In our patient, negative ANA and anti‐dsDNA antibodies and normal complement studies made active SLE unlikely at the time of diagnosis. Repeat autoimmune serologies obtained several weeks after hospitalization remained negative. Nevertheless, because KFD has been reported to precede or coexist with SLE, continued clinical surveillance for subsequent autoimmune disease is warranted [11, 12]. Longer‐term rheumatologic follow‐up was not available in this case. Key distinguishing features of KFD and important infectious, malignant, and autoimmune causes of cervical lymphadenopathy are summarized in Table 2.

**TABLE 2:** Key features in the differential diagnosis of cervical lymphadenopathy.

Diagnosis	Typical features	Distinguishing features
Kikuchi–Fujimoto disease [2, 4, 5]	• Acute to subacute • Usually unilateral cervical lymphadenopathy • Tender/painful nodes, often with fever	• Leukopenia may occur • Necrotizing lymphadenitis with histiocytes • Characteristic absence of neutrophils

Bacterial lymphadenitis [13]	• Acute • Typically unilateral • Tender nodes with fever ± overlying erythema	• Neutrophilic/suppurative inflammation • Cultures may identify bacterial pathogen • Clinical improvement with appropriate antibiotics

Lymphoma [13]	• Usually subacute/chronic • Localized or generalized lymphadenopathy • Typically painless	• Firm/rubbery nodes • Abnormal lymphoid population on histopathology/flow cytometry

SLE lymphadenitis [11, 12]	• May be localized or generalized • May accompany systemic manifestations • Fever and lymphadenopathy	• Positive autoimmune serologies • Hypocomplementemia may occur • Hematoxylin bodies may help distinguish from KFD

As KFD is considered self‐limited, management consists primarily of supportive care, with nonsteroidal anti‐inflammatory drugs commonly used for symptom relief, though corticosteroids and immunomodulatory therapies may be considered in severe or recurrent disease [14–16]. Our patient experienced resolution of fever and improvement in cervical swelling temporally following two doses of IV ceftriaxone despite an extensive infectious evaluation that was ultimately unrevealing. Following definitive diagnosis, she was managed supportively with naproxen for residual inflammation.

This case illustrates how the clinical course of pediatric KFD can complicate diagnostic reasoning when its manifestations overlap with infection and malignancy. In our patient, the prolonged course despite multiple antibiotic regimens, necrotic lymphadenopathy, apparent clinical improvement temporally associated with antimicrobial therapy, and initially nondiagnostic minimally invasive tissue sampling collectively obscured the diagnosis. Recognition that clinical improvement during empiric antimicrobial therapy may coincide with the natural evolution of a self‐limited disease is important to avoid overattributing treatment response to an infectious etiology. When cervical lymphadenopathy persists or recurs despite appropriate antimicrobial therapy and infectious evaluations remain unrevealing, KFD should be considered, and excisional lymph node biopsy pursued when less invasive sampling is nondiagnostic.

## 4. Conclusion

KFD is a rare, self‐limited disease of unknown etiology that causes subacute cervical lymphadenopathy and systemic symptoms in children. It should be considered in pediatric patients who present with persistent cervical lymphadenopathy refractory to antibiotic therapy, particularly when associated with cytopenias, transaminitis, or nondiagnostic infectious workup. This case highlights the diagnostic challenges of KFD, including its overlap with infectious, malignant, and autoimmune etiologies, as well as the limited utility of FNA. Excisional lymph node biopsy remains the gold standard for diagnosis and may help avoid unnecessary prolonged treatment and extensive diagnostic evaluation. Continued follow‐up is important given the reported association between KFD and SLE.

## Funding

No funding was received for this study.

## Conflicts of Interest

The authors declare no conflicts of interest.

## Data Availability

Data sharing is not applicable to this article as no datasets were generated or analyzed during the current study.
